# Effects of changing the timing of warfarin administration in combination with fluconazole on prolongation of the PT-INR: a case report

**DOI:** 10.1186/s40780-023-00279-w

**Published:** 2023-04-01

**Authors:** Hayato Akamatsu, Hiroo Nakagawa, Ichiro Matsumaru, Junya Hashizume, Hitomi Harasawa, Yukinobu Kodama, Takashi Miura, Kaname Ohyama

**Affiliations:** 1 Department of Hospital Pharmacy, Nagasaki University Hospital, 1-7-1 Sakamoto, Nagasaki, 852-8501 Japan; 2 Department of Cardiovascular Surgery, Nagasaki University Hospital, 1-7-1 Sakamoto, Nagasaki, 852-8501 Japan; 3 Department of Medical Safety, Nagasaki University Hospital, 1-7-1 Sakamoto, Nagasaki, 852-8501 Japan

**Keywords:** Fluconazole, Warfarin, PT-INR, Drug-drug interaction, Timing of administration

## Abstract

**Background:**

Fluconazole (FLCZ) inhibits cytochrome P450 (CYP) 2C9, 2C19, and 3A4 and has a drug-drug interaction that potentiates the effects of warfarin and prolong the prothrombin time-international normalized ratio (PT-INR). Although a drug-drug interaction have been reported between FLCZ and warfarin, the effects of the timing of their administration on this interaction have not yet been investigated.

**Case presentation:**

A female patient in her 30s with Marfan syndrome had undergone the Bentall procedure with a mechanical valve and total arch replacement for acute aortic dissection Stanford A type and rupture of the ascending aorta. Warfarin was administered to prevent thromboembolism. She was hospitalized 1 year ago for graft infection caused by *Candida albicans*, and treatment with FLCZ was initiated. She received FLCZ 200 mg once a day in the morning and warfarin 1.75 mg once a day in the evening, and the PT-INR remained stable at approximately 2.0 and within the therapeutic range. However, 42 days after changing the timing of administration of warfarin from evening to morning, the PT-INR was prolonged by approximately 3-fold to 6.25. The PT-INR then decreased to the previous level by changing the timing of administration of warfarin from morning to evening.

**Conclusions:**

The timing of administration of FLCZ and warfarin may affect the magnitude of drug-drug interaction.

## Background

Fluconazole (FLCZ) is an azole antifungal drug that is primarily used to treat infections caused by *Candida* and *Cryptococcus* species. Warfarin is an anticoagulant that is used to prevent and treat thromboembolism following valve replacement surgery, atrial fibrillation, and pulmonary embolism. FLCZ potentiates the effects of warfarin by inhibiting the drug-metabolizing enzyme cytochrome P450 (CYP) 2C9, resulting in a drug-drug interaction that prolongs the prothrombin time-international normalized ratio (PT-INR). Although the drug-drug interaction between FLCZ and warfarin has been examined [1], the effects of the timing of their administration on this drug-drug interaction currently remain unclear [2–8]. We herein present a case in which the PT-INR was markedly prolonged after changing the timing of the administration of FLCZ and warfarin from morning and evening, respectively, to simultaneous morning administration.

## Case presentation

The patient was a female in her 30s with Marfan syndrome who had undergone the Bentall procedure with a mechanical valve and total arch replacement for acute aortic dissection Stanford A type and rupture of the ascending aorta. She was administered warfarin to prevent thromboembolism. The dose of warfarin was 5–7 mg per day, which controlled the PT-INR at 1.8–2.4. One year ago, the patient underwent total arch replacement surgery for a suspected prosthetic vascular infection of unknown origin, but developed a postoperative aortoesophageal fistula and mediastinitis caused by *Candida albicans*. After continuous lavage and endoscopic-assisted esophagectomy (esophagostomy and gastrostomy), the patient was discharged home with infection controlled by continuous suction therapy and fluconazole administered via gastrostomy.

**Fig. 1:**
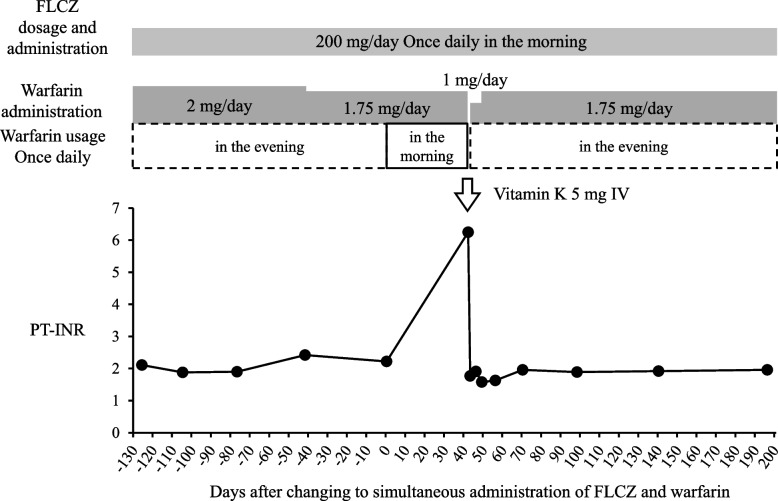
FLCZ and warfarin dosages and the PT-INR before and after the change in the timing of warfarin administration. *FLCZ* fluconazole, *PT-INR* prothrombin time-international normalized ratio, *IV*: intravenous injection, Day 0 was defined as the day when FLCZ and warfarin were changed to simultaneous administration

After discharge from the hospital, the patient visited the outpatient clinic every month. Table 1 shows the medications the patient was taking during outpatient visits. Capsules were decapsulated and tablets were crushed and administered via gastrostomy as follows: FLCZ 200 mg once a day in the morning and warfarin 1.75 mg once a day in the evening. The PT-INR was stable at approximately 2.0 and within the therapeutic range (Fig. 1). In an outpatient visit, the attending physician did not change the dosage of warfarin, but changed the timing of its administration to the morning, which was concurrent with FLCZ, in order to improve medication compliance. In an outpatient visit 42 days later, there was no apparent bleeding event; however, the PT-INR was prolonged by approximately 3-fold to 6.25. Therefore, the patient was urgently admitted to the hospital on the same day. Warfarin was discontinued, intravenous vitamin K 5 mg was administered, and the PT-INR decreased to 1.77 the day after admission. The administration of warfarin 1 mg once a day in the evening was resumed and the patient was discharged on the second day of admission. In an outpatient visit 6 days later, a decrease in the PT-INR to 1.58 was observed; therefore, the dose of warfarin was increased to 1.75 mg once a day in the evening, the same dose as that before the marked increase in the PT-INR. Thereafter, the PT-INR decreased to the original level of approximately 2.0 and remained stable for more than 6 months.

**Table 1:** Medications taken in outpatient visits

Drug administered	Dose	Frequency and Timing
Warfarin Granules	1.75 mg	once a day in the evening (before the change in the timing of administration)
Fluconazole capsule	200 mg	once a day in the morning
Bisoprolol fumarate tablet	2.5 mg	once a day in the morning
Bonoprazan tablet	10 mg	once a day in the morning
Metoclopramide tablet	5 mg	once when nauseous
RACOL-NF^Ⓡ^ Semi Solid for Enteral Use	2 packs	3 times a day in the morning, noon, and evening

## Discussion and conclusions

We encountered a case in which the PT-INR was markedly prolonged by the combination of FLCZ and warfarin when both drugs with different dosing timings were switched to simultaneous administration. Since this patient had undergone mechanical valve replacement and was at high risk of thromboembolism, permanent anticoagulation with warfarin was recommended [9]. In the period after the Bentall procedure with a mechanical valve 5 years ago and before starting the combination of FLCZ, the PT-INR was controlled at 1.8–2.4 with warfarin 5 to 7 mg per day in the evening. After the combination of warfarin with FLCZ 200 mg, the PT-INR was maintained at a dose of 1.75 mg of warfarin. The blood half-life of warfarin is 36.3 ± 3.5 h, as previously reported by Vesell et al. [10], while that of FLCZ is approximately 31 h [11]. Since both drugs are administered once a day and the patient was in a steady state of continuous daily administration, a drug-drug interaction between the two drugs had clearly occurred. Previous studies reported the prolongation of the PT-INR and gastrointestinal hemorrhage [2], epistaxis, gingival hemorrhage, mottling of the extremities [4], intraocular hemorrhage [6], and retropharyngeal hematoma with marked narrowing of the glottis [8]. However, the timing of administration of the two drugs was not described, and the effects of their timing on the PT-INR was not known. Warfarin exerts its anticoagulant effect by inhibiting the biosynthesis of vitamin K-dependent coagulation factors II (prothrombin), VII, IX, and X in the liver. Therefore, the action of warfarin is affected by the intake of vitamin K, and the PT-INR may change depending on its amount in the diet [12]. However, since this patient had undergone esophagectomy, nutrition was provided by tube feeding through gastrostomy and not by oral intake. The type and dosage of tube feedings during the periods before and after the PT-INR was markedly prolonged remained unchanged, suggesting that food and drink did not affect the PT-INR. There were also no changes in concomitant medications other than FLCZ and warfarin. The patient self-administered her medications; she was in her 30s and was well-adherent. There was no possibility of warfarin misadministration based on the remaining medications she had brought with her upon admission. Because warfarin is completely absorbed in the stomach and proximal small intestine [13], its absorption is unlikely to be affected by the administration through a gastrostomy. Furthermore, when a marked increase in the PT-INR was observed, vitamin K was administered and the PT-INR decreased. The warfarin dosage was subsequently returned to 1.75 mg once a day in the evening before the marked increase in the PT-INR, and the PT-INR decreased to the original level of approximately 2.0. These results indicate that the only cause of the markedly prolonged PT-INR was the change from the different dosing timings of both drugs to simultaneous dosing under the combination of FLCZ and warfarin.

The markedly prolonged PT-INR may be attributed to the simultaneous administration of FLCZ and warfarin, strongly inhibiting the metabolism of S-warfarin, which exhibits strong anticoagulant activity among the optical isomers of warfarin (S-warfarin and R-warfarin), due to the inhibitory effects of FLCZ on CYP2C9, 2C19, and 3A4 in the liver and gastrointestinal tract. Other diurnal variations in the activities of CYP3A and CYP1A2, which are involved in R-warfarin metabolism, were also observed, suggesting that differences in the timing of warfarin administration affected R-warfarin metabolism. In addition, the variation in hepatic CYP3A activity was observed in the range of 10% higher than average around 15:00 and 10% lower than average around 3:00, but their magnitude was reported to be small and of no clinical significance for drug therapy [14]. There is also a report that CYP1A2 activity differs between morning and evening [15], but its significance in clinical practice is unclear. Other factors may be involved in a complex manner, and the elucidation of these mechanism remains a future challenge.

In the case of the combination of FLCZ and warfarin, the timing of drug administration as well as drug-drug interactions need to be considered. However, since this is a single case report, the further accumulation of cases is needed.

## Data Availability

Not applicable.
